# Supplementary material for: Functional annotation signatures of disease susceptibility loci improve SNP association analysis
Source: BMC Genomics. 2014 May 24;15(1):398. doi: 10.1186/1471-2164-15-398 (PMC4041996; doi:10.1186/1471-2164-15-398)
Supplement: Supplementary file 1 — Additional file 1: Supplemental table and figures. (PDF 435 KB) [file 12864_2013_6087_MOESM1_ESM.pdf]

Supplemental Table and Figures
Functional Annotation Signatures of Disease Susceptibility Loci Improve SNP Association Analysis

Annotation	Location	Date◇
GWAS Catalog	gwasCatalog.txt	11/15/10
Affymetrix 500K Set	snpArrayAffy250{Nsp, Sty}.txt	08/02/07
Illumina 550K Set	snpArrayIllumina550.txt	08/02/07
dbSNP 130	snp130.txt	09/20/09
HapMap Rel27	hapmapSnpsCEU.txt	07/11/07
HapMap Rel27 LD†	ld.chr{1..22}.CEU.txt	02/09
Broad GM12878 H3K27ac	wgEncodeBroadChipSeqSignalGm12878H3k27ac.txt.gz	06/22/10
Broad GM12878 H3K4me1	wgEncodeBroadChipSeqSignalGm12878H3k4me1.txt.gz	06/22/10
Broad GM12878 H3K4me3	wgEncodeBroadChipSeqSignalGm12878H3k4me3.txt.gz	06/22/10
Broad H1hesc H3K4me1	wgEncodeBroadChipSeqSignalH1hescH3k4me1.txt.gz	06/22/10
Broad H1hesc H3K4me3	wgEncodeBroadChipSeqSignalH1hescH3k4me3.txt.gz	06/22/10
Broad HepG2 H3K27ac	wgEncodeBroadChipSeqSignalHepg2H3k27ac.txt.gz	06/22/10
Broad HepG2 H3K4me3	wgEncodeBroadChipSeqSignalHepg2H3k4me3.txt.gz	06/22/10
Broad HMEC H3K27ac	wgEncodeBroadChipSeqSignalHmecH3k27ac.txt.gz	06/22/10
Broad HMEC H3K4me1	wgEncodeBroadChipSeqSignalHmecH3k4me1.txt.gz	06/22/10
Broad HMEC H3K4me3	wgEncodeBroadChipSeqSignalHmecH3k4me3.txt.gz	06/22/10
Broad HSMM H3K27ac	wgEncodeBroadChipSeqSignalHsmmH3k27ac.txt.gz	06/22/10
Broad HSMM H3K4me1	wgEncodeBroadChipSeqSignalHsmmH3k4me1.txt.gz	06/22/10
Broad HSMM H3K4me3	wgEncodeBroadChipSeqSignalHsmmH3k4me3.txt.gz	06/22/10
Broad HUVEC H3K27ac	wgEncodeBroadChipSeqSignalHuvecH3k27ac.txt.gz	06/22/10
Broad HUVEC H3K4me1	wgEncodeBroadChipSeqSignalHuvecH3k4me1.txt.gz	06/22/10
Broad HUVEC H3K4me3	wgEncodeBroadChipSeqSignalHuvecH3k4me3.txt.gz	06/22/10
Broad K562 H3K27ac	wgEncodeBroadChipSeqSignalK562H3k27ac.txt.gz	06/22/10
Broad K562 H3K4me1	wgEncodeBroadChipSeqSignalK562H3k4me1.txt.gz	06/22/10
Broad K562 H3K4me3	wgEncodeBroadChipSeqSignalK562H3k4me3.txt.gz	06/22/10
Broad NHEK H3K27ac	wgEncodeBroadChipSeqSignalNhekH3k27ac.txt.gz	06/22/10
Broad NHEK H3K4me1	wgEncodeBroadChipSeqSignalNhekH3k4me1.txt.gz	06/22/10
Broad NHEK H3K4me3	wgEncodeBroadChipSeqSignalNhekH3k4me3.txt.gz	06/22/10
Broad NHLF H3K27ac	wgEncodeBroadChipSeqSignalNhlfH3k27ac.txt.gz	06/22/10
Broad NHLF H3K4me1	wgEncodeBroadChipSeqSignalNhlfH3k4me1.txt.gz	06/22/10
Broad NHLF H3K4me3	wgEncodeBroadChipSeqSignalNhlfH3k4me3.txt.gz	06/22/10
Caltech Rep1 GM12878 Long PolyA BB1 2x75	wgEncodeCaltechRnaSeqRawSignalRep1Gm12878CellLongpolyaBb12x75.txt.gz	12/20/09
Caltech Rep1 H1hesc PAP BB2R 2x75	wgEncodeCaltechRnaSeqRawSignalRep1H1hescCellPapBb2R2x75.txt.gz	06/14/10
Caltech Rep1 HUVEC PAP BB2R 2x75	wgEncodeCaltechRnaSeqRawSignalRep1HuvecCellPapBb2R2x75.txt.gz	06/14/10
Caltech Rep1 K562 Long PolyA BB1 2x75	wgEncodeCaltechRnaSeqRawSignalRep1K562CellLongpolyaBb12x75.txt.gz	12/20/09
Caltech Rep1 NHEK PAP BB2R 2x75	wgEncodeCaltechRnaSeqRawSignalRep1NhekCellPapBb2R2x75.txt.gz	06/15/10
Caltech Rep2 HepG2 PAP BB2R 2x75	wgEncodeCaltechRnaSeqRawSignalRep2Hepg2CellPapBb2R2x75.txt.gz	06/14/10
DNase I Hypersensitivity Clusters	wgEncodeRegDnaseClustered.txt.gz	08/15/10
TFBS Clustered ChIP-seq	wgEncodeRegTfbsClustered.txt.gz	08/15/10
PhyloP 28–Way Base Cons	phyloP28way.txt.gz	11/30/08
PhyloP 28-Way Base Cons Plac Mammal	phyloP28wayPlacMammal.txt.gz	11/30/08
PhyloP 44-Way Base Cons	phyloP44wayAll.txt.gz	02/02/09
PhyloP 44-Way Base Cons Plac Mammal	phyloP44wayPlacMammal.txt.gz	02/02/09
PhyloP 44-Way Base Cons Primate	phyloP44wayPrimate.txt.gz	02/02/09
ORegAnno	oreganno.txt.gz	07/31/08
DGV Indel‡	indel.hg18.v10.nov.2010.txt	11/10
DGV Variation‡	variation.hg18.v10.nov.2010.txt	11/10
PolyPhen–2	pph2-full.hg18.txt	06/26/13
RegulomeDB	RegulomeDB.dbSNP132.Category[1-7].txt	06/25/13

All files downloaded from `http://hgdownload.cse.ucsc.edu/goldenPath/hg18/database`, except as noted.
†`http://hapmap.ncbi.nlm.nih.gov/downloads/ld_data/2009-04_rel27` ‡`http://projects.tcag.ca/variation/downloads` ◇For UCSC, date stamp is for associated SQL file, e.g. gwasCatalog.sql.

Supplemental Table 1: Summary of data sources.

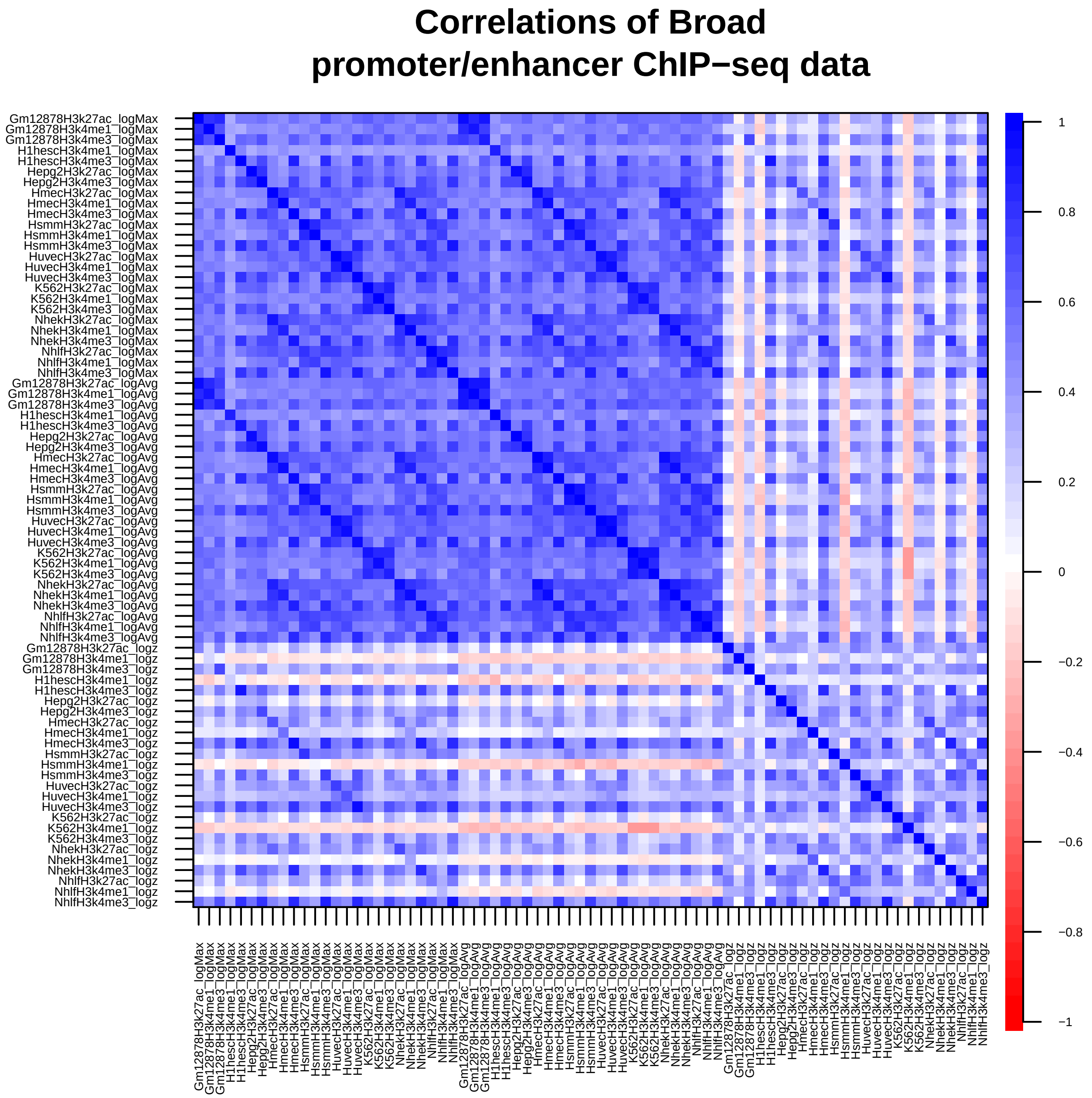

Supplemental Figure 1: Heat map of the correlation matrix of of the 75 Broad ChIP–seq variables used in the analysis. These were reduced to the top 18 principal components explaining 90% of variability in these measures.

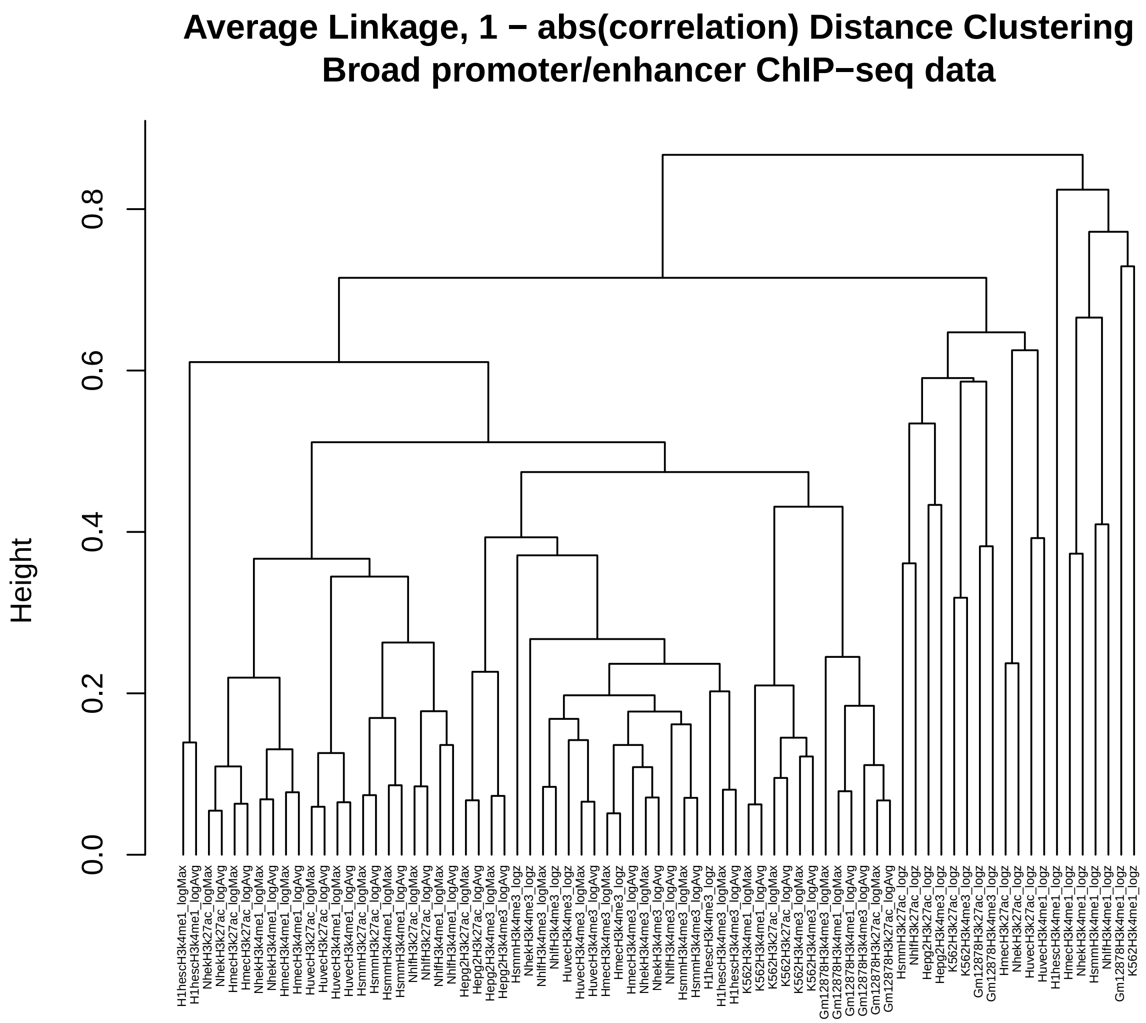

Supplemental Figure 2: Dendrogram depicting an average linkage, 1 - absolute correlation distance clustering of the 75 Broad ChIP–seq variables used in the analysis.

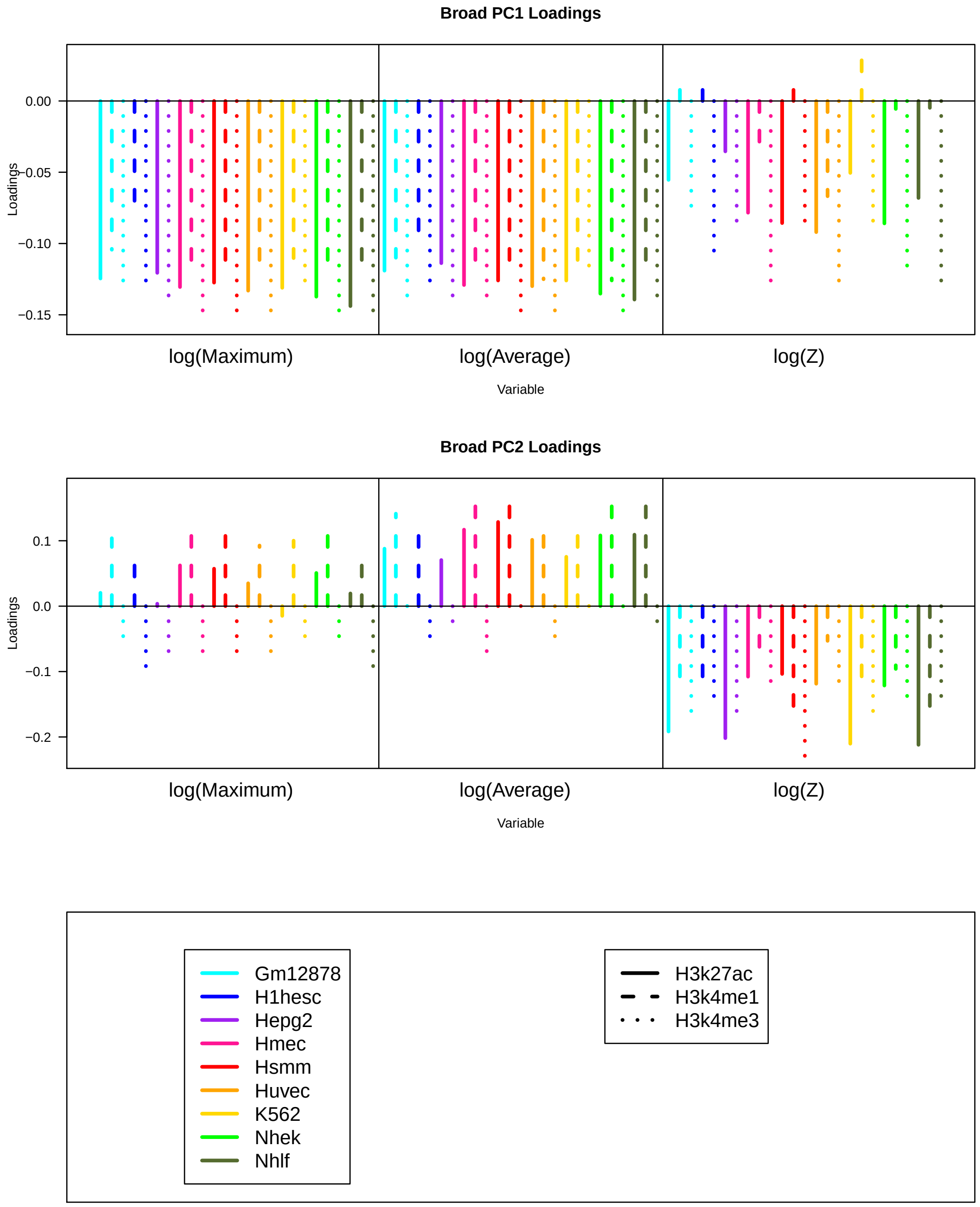

Supplemental Figure 3: Plot of the loadings (weights in the linear combinations) for the most highly associated Broad promoter/enhancer ChIP–seq principal components (PCs) as they depend on histone modification, cell line and summary statistic.

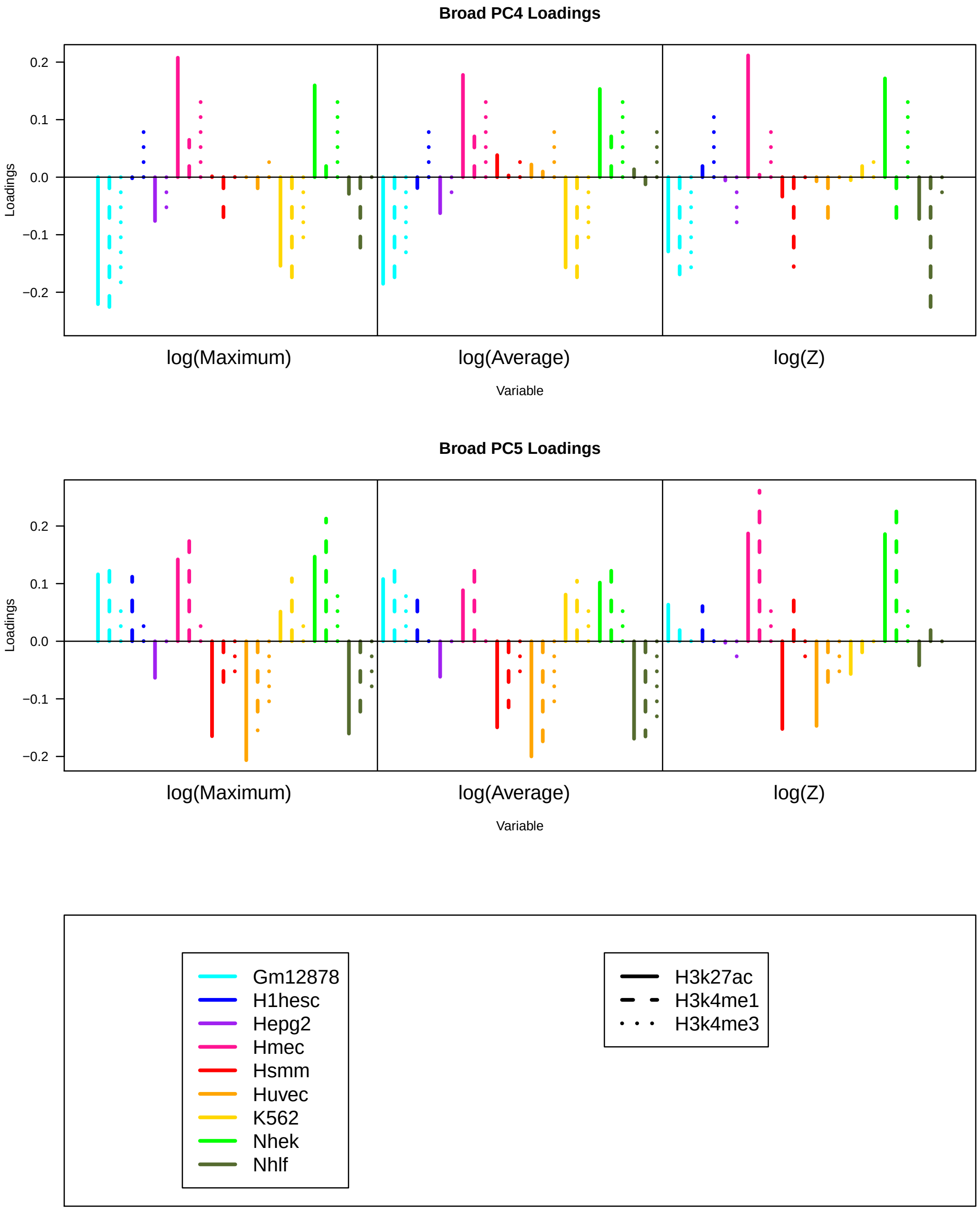

Supplemental Figure 3, Continued: Plot of the loadings (weights in the linear combinations) for the most highly associated Broad promoter/enhancer ChIP–seq principal components (PCs) as they depend on histone modification, cell line and summary statistic.

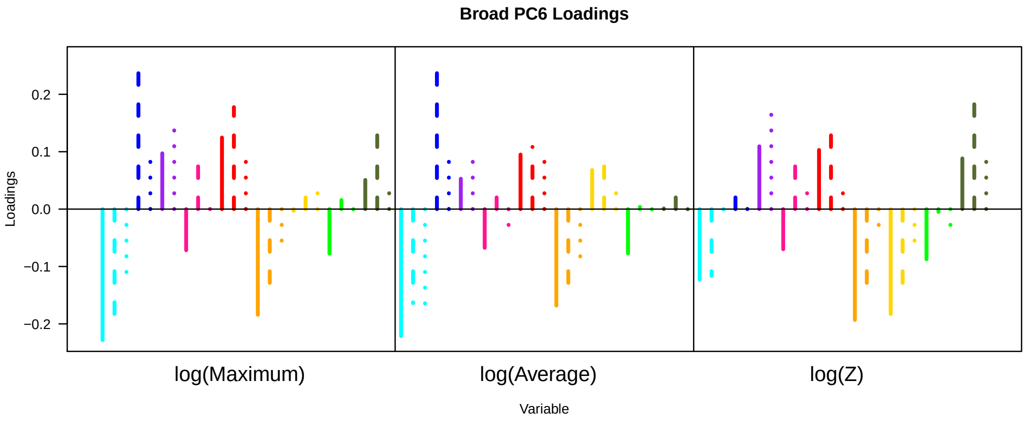

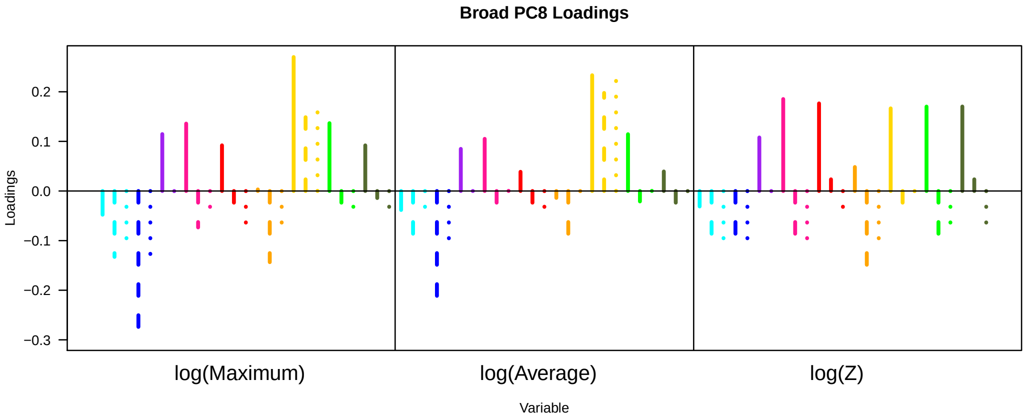

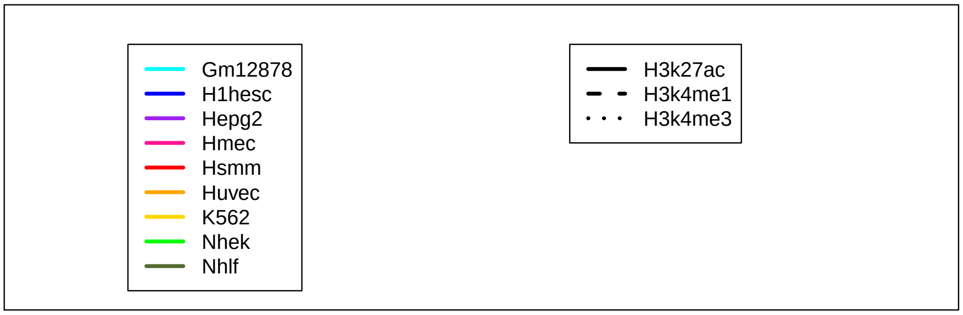

Supplemental Figure 3, Continued: Plot of the loadings (weights in the linear combinations) for the most highly associated Broad promoter/enhancer ChIP–seq principal components (PCs) as they depend on histone modification, cell line and summary statistic.

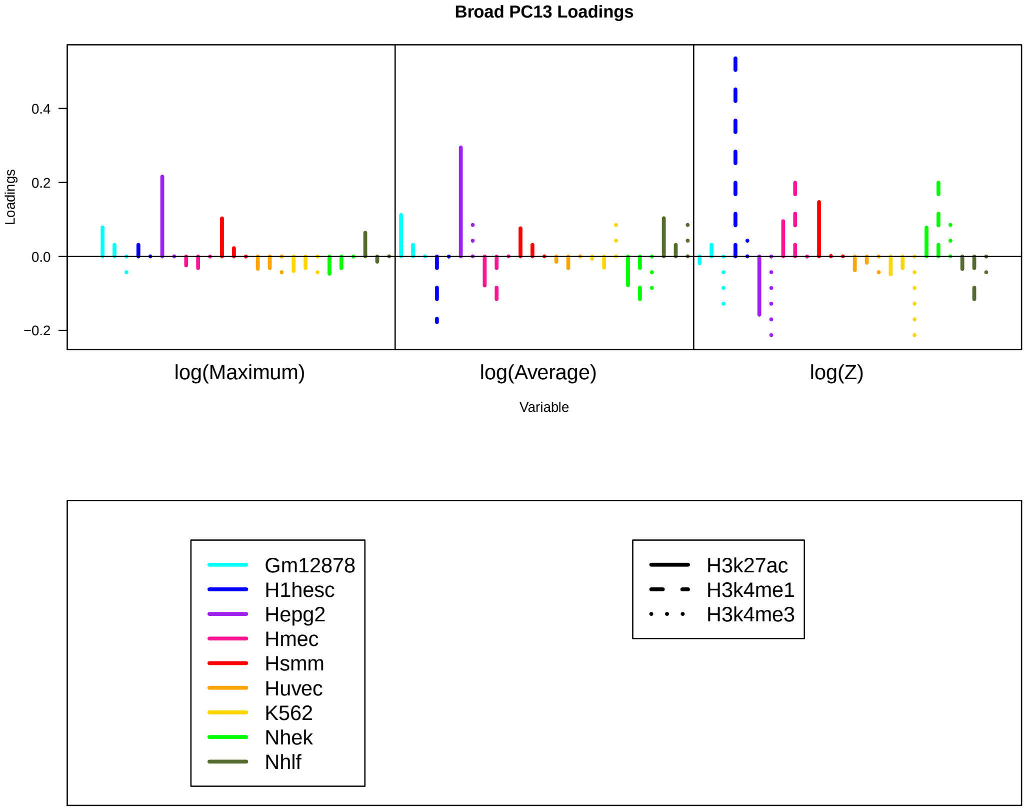

Supplemental Figure 3, Continued: Plot of the loadings (weights in the linear combinations) for the most highly associated Broad promoter/enhancer ChIP–seq principal components (PCs) as they depend on histone modification, cell line and summary statistic.

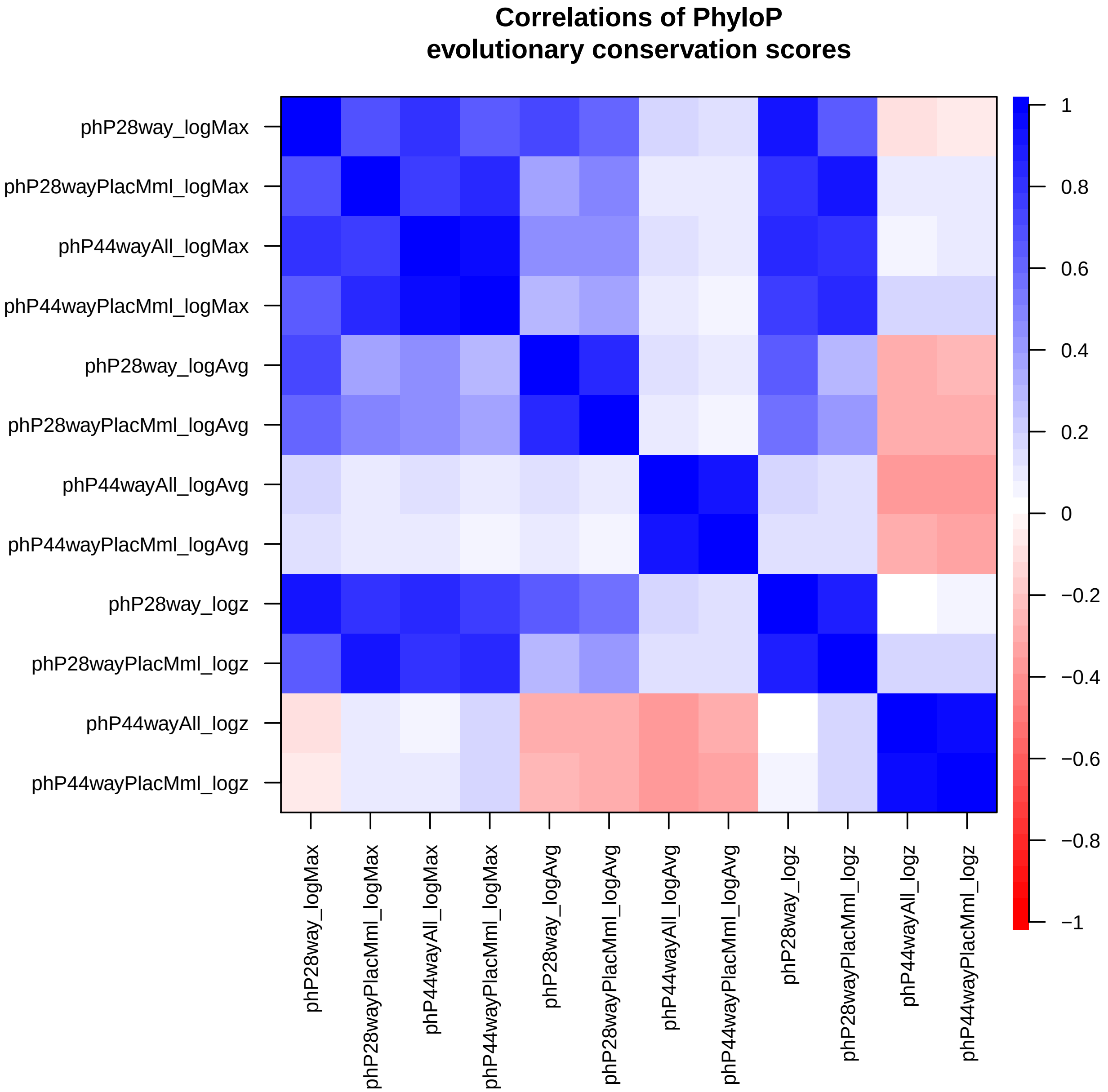

Supplemental Figure 4: Heat map of the correlation matrix of of the 12 PhyloP evolutionary conservation score variables used in the analysis. These were reduced to the top 4 principal components explaining 90% of variability in these measures.

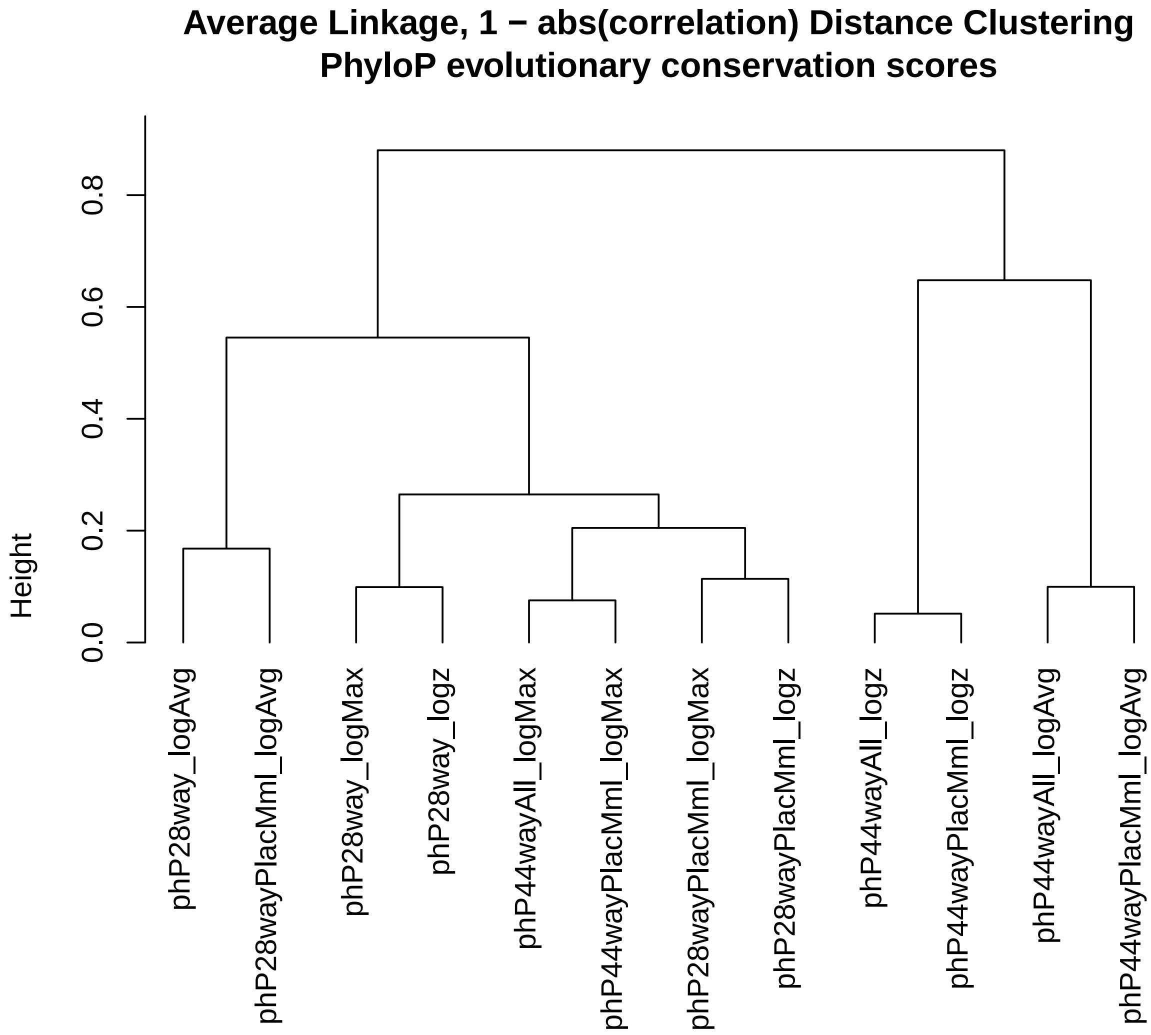

Supplemental Figure 5: Dendrogram depicting an average linkage, 1 - absolute correlation distance clustering of the 12 PhyloP evolutionary conservation score variables used in the analysis.

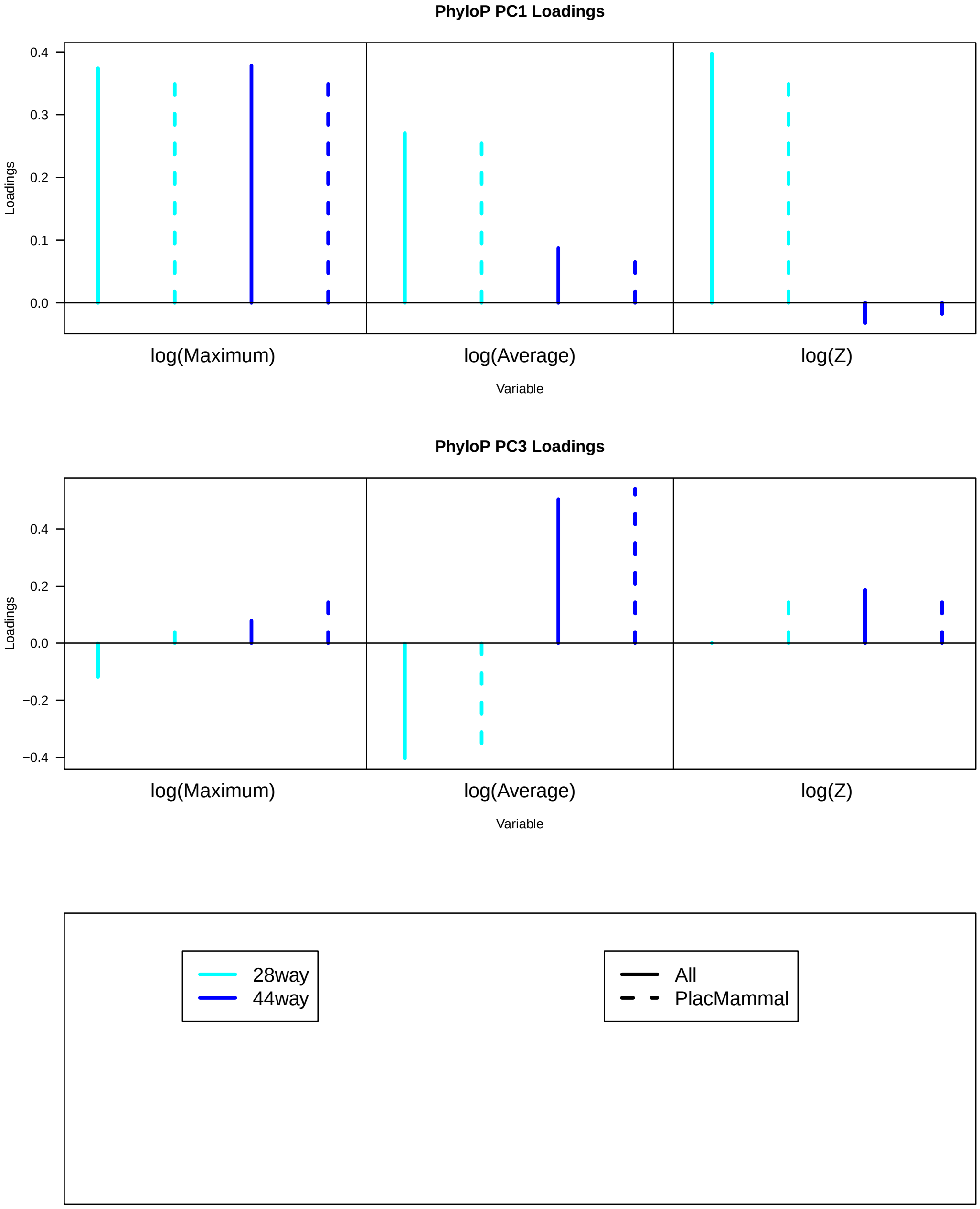

Supplemental Figure 6: Plot of the loadings (weights in the linear combinations) for the most highly associated sequence conservation principal components (PCs) as they depend on number of species, depth of alignment and summary statistics.

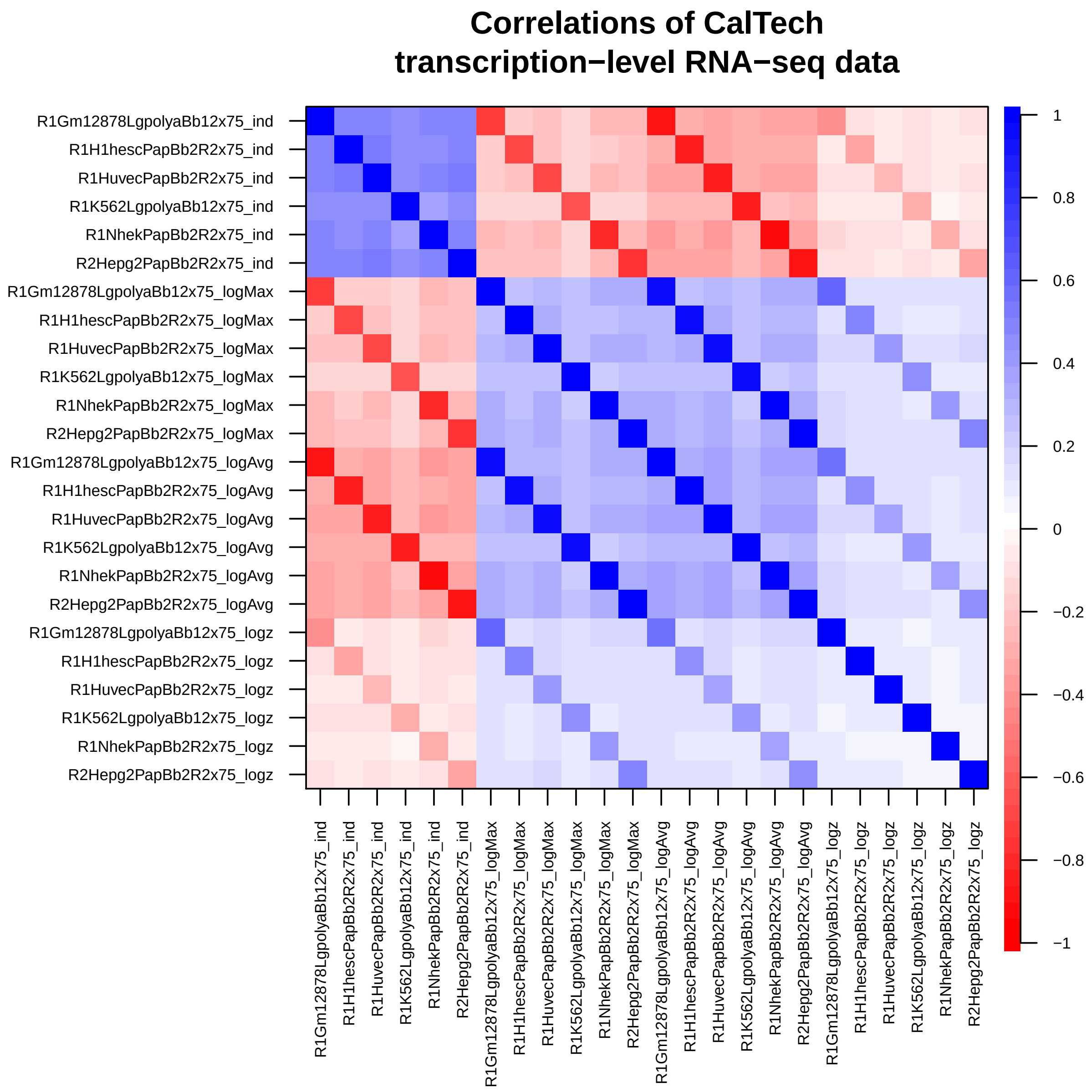

Supplemental Figure 7: Heat map of the correlation matrix of the 24 Caltech RNA–seq variables used in the analysis. These were reduced to the top 11 principal components explaining 92% of variability in these measures.

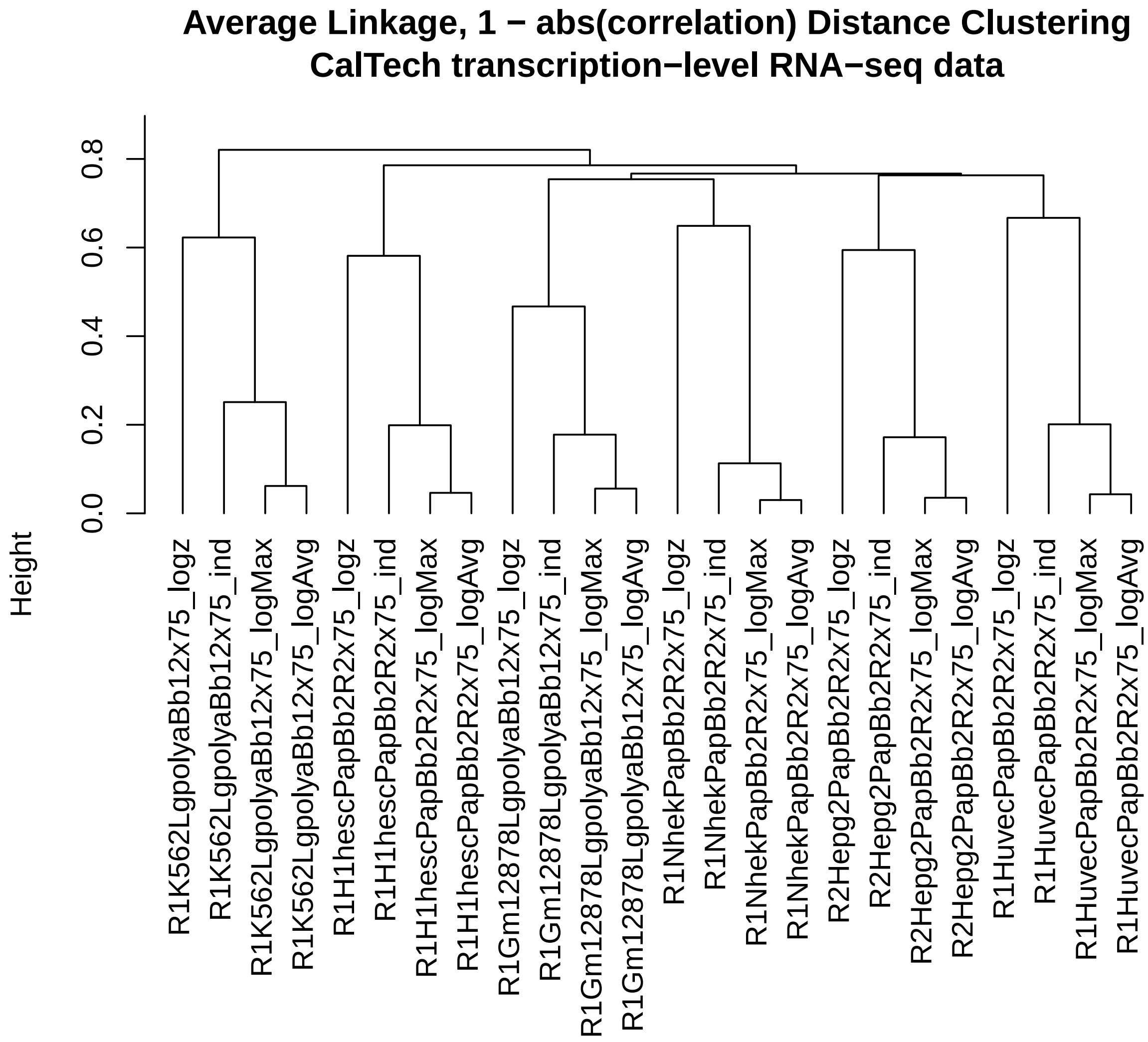

Supplemental Figure 8: Dendrogram depicting an average linkage, 1 - absolute correlation distance clustering of the 24 Caltech RNA–seq variables used in the analysis.

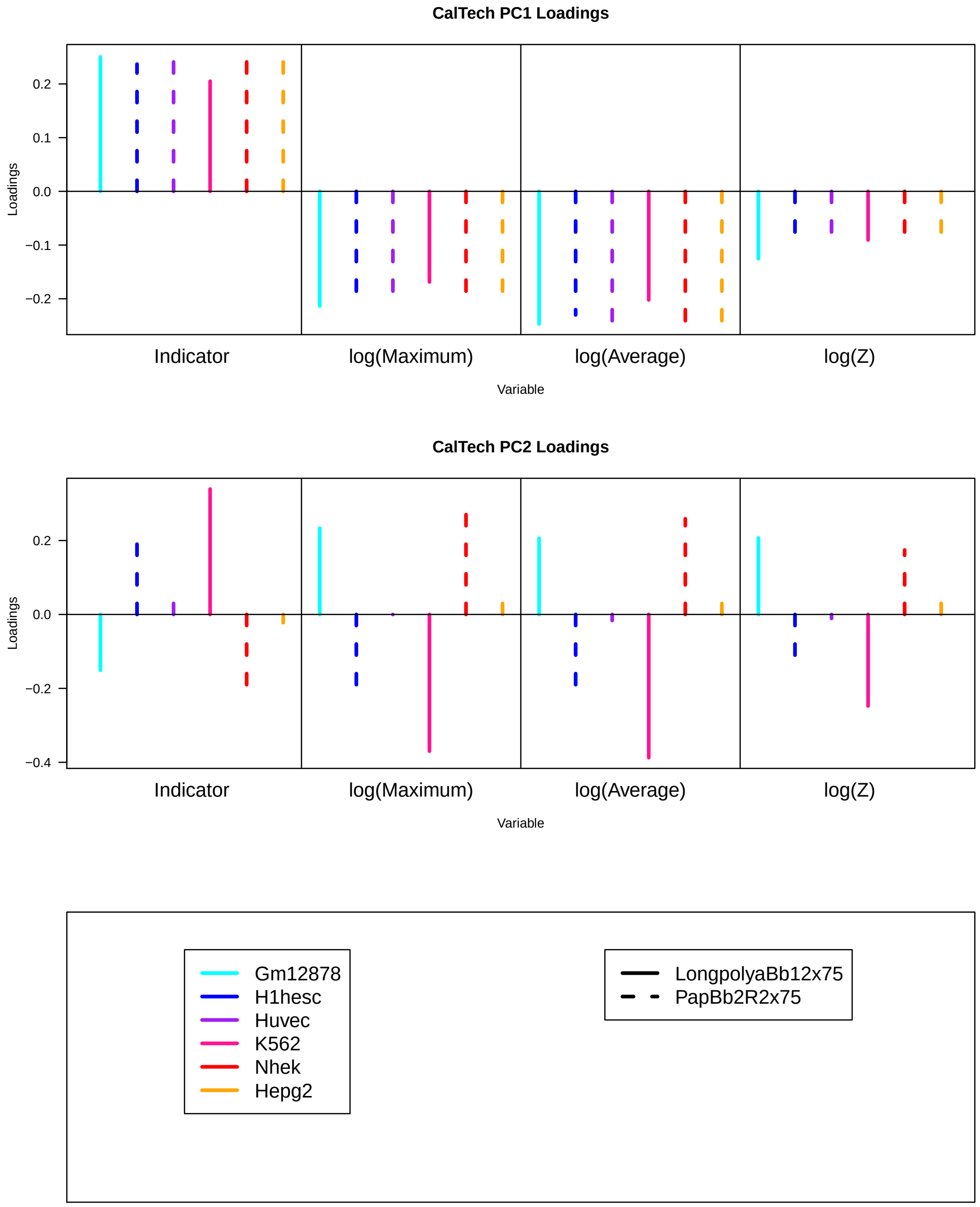

Supplemental Figure 9: Plot of the loadings (weights in the linear combinations) for the most highly associated CalTech RNA–seq principal components (PCs) as they depend on cell line and summary statistic.

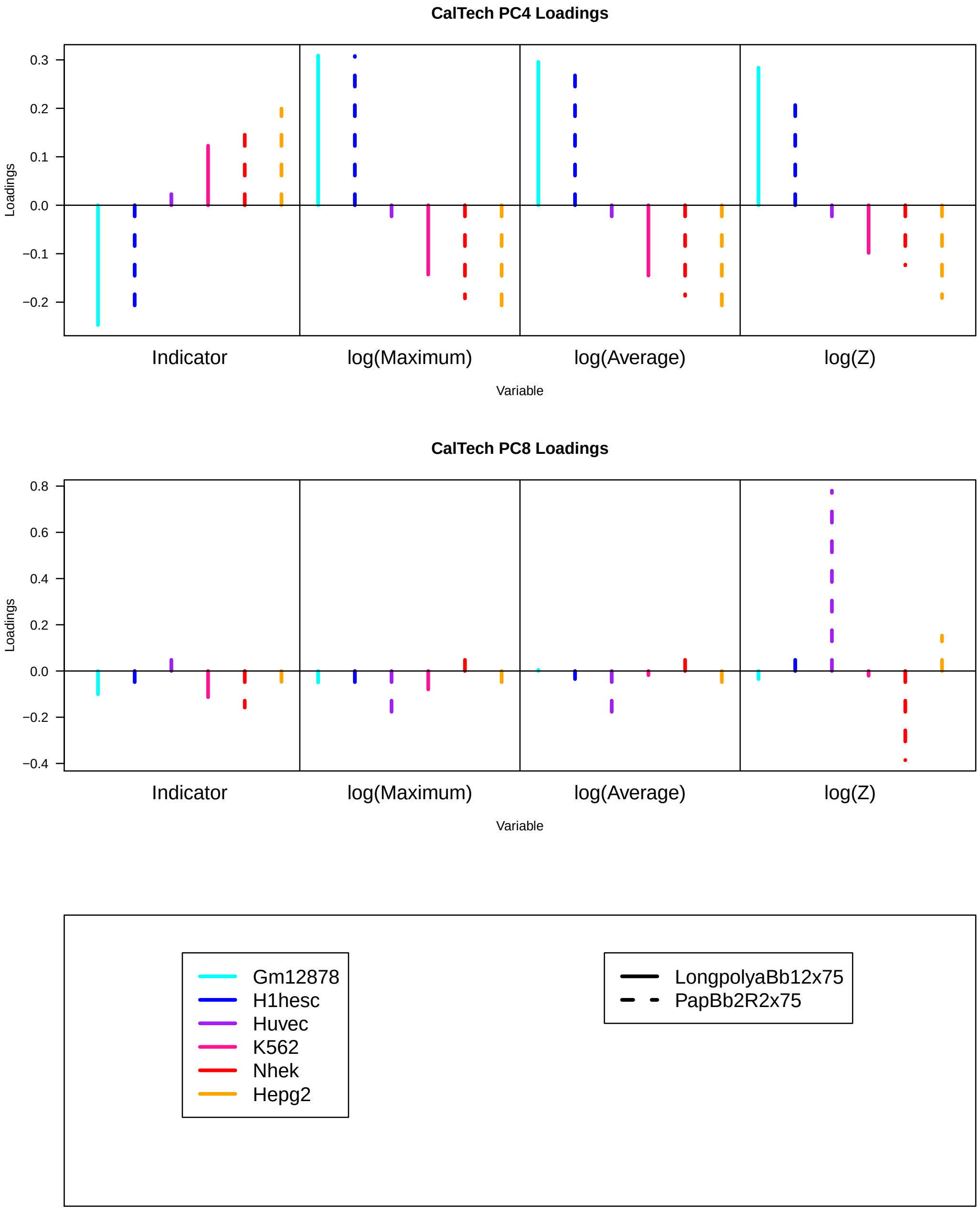

Supplemental Figure 9, Continued: Plot of the loadings (weights in the linear combinations) for the most highly associated CalTech RNA–seq principal components (PCs) as they depend on cell line and summary statistic.

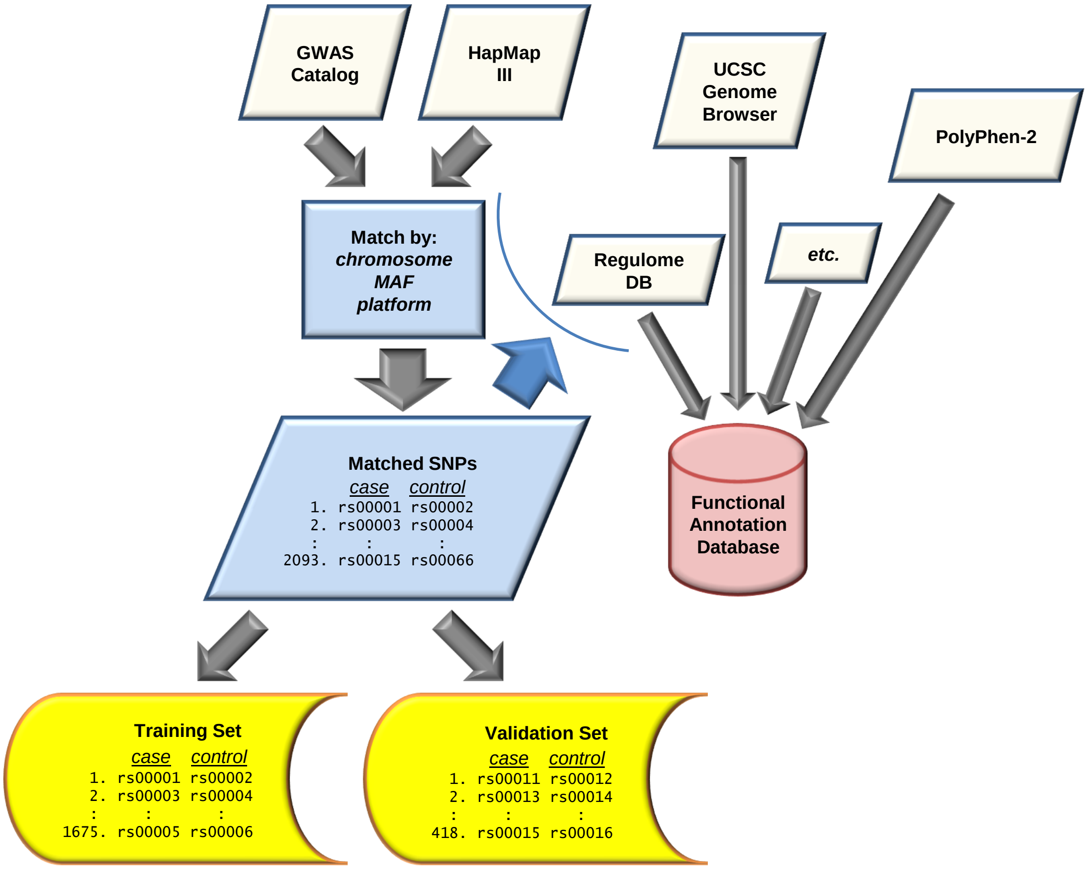

Supplemental Figure 10: Construction of data sets and functional annotation database. Case SNPs from the GWAS Catalog are matched with control SNPs from HapMap III to generate training and validation sets. The matched SNP IDs and their locations are used to interrogate several online databases. These results are merged to build the functional annotation database.
